# The Cost of Locally Advanced Cervical Cancer in Thailand: an Empirical Study for Economic Analysis 

**DOI:** 10.31557/APJCP.2021.22.10.3171

**Published:** 2021-10

**Authors:** Kanyarat Katanyoo, Arthorn Riewpaiboon, Usa Chaikledkaew, Montarat Thavorncharoensap

**Affiliations:** 1 *Social, Economic and Administrative Pharmacy (SEAP) Graduate Program, Faculty of Pharmacy, Mahidol University, Bangkok, Thailand. *; 2 *Radiation Oncology Unit, Department of Radiology, Faculty of Medicine Vajira Hospital, Navamindradhiraj University, Bangkok, Thailand. *; 3 *Social and Administrative Pharmacy Division, Department of Pharmacy, Faculty of Pharmacy, Mahidol University, Bangkok, Thailand. *

**Keywords:** Cervical cancer, cost of illness, economic burden, Thailand

## Abstract

**Objective:**

To evaluate cost of illness of locally advanced cervical cancer patients from societal perspective in three scenarios including completely cured without severe late side effects (S1), completely cured with late grade 3-4 gastrointestinal side effects (S2.1) or genitourinary side effects (S2.2), and disease recurrence and death (S3).

**Methods:**

The incidence-based approach was conducted. The cost was calculated for 5-year time horizon starting for the treatment initiation. Direct medical costs were extracted from hospital database. Cost of using two-dimensional technique and three-dimensional conformal radiation therapy were calculated separately. Direct non-medical costs and indirect costs in terms of productivity loss were based on actual expenses from the interview of 194 locally advanced cervical cancer patients from two tertiary hospitals in Bangkok, during June to December 2019. All costs were converted to US dollar in 2019 values.

**Results:**

For 5 years, cost of illness per patient for using two-dimensional technique and three-dimensional conformal radiation therapy were US $8,391 and US $10,418 for S1, US $18,018 and US $20,045 for S2.1, US $17,908 and US $19,936 for S2.2, and US $61,076 and US $63,103 for S3, respectively. The economic burden for newly diagnosed locally advanced cervical cancer patients in Thailand in 2018 was approximately US $129 million and US $131 million for using two-dimensional technique and three-dimensional conformal radiation therapy, respectively. Cost from S3 accounted for 70% of all total cost. Premature death was the most important cost driver of cost of illness accounted for 64 % of the total cost estimates.

**Conclusions:**

Cost of illness of locally advanced cervical cancer patients produced significant economic burden from societal perspective. Disease recurrence and early death from cancer was the most influential cause of this burden.

## Introduction

Cervical cancer causes significant burden in low and middle income countries (Bray et al., 2018). In Thailand, age-standardized incidence of cervical cancer was 16.2 per 100,000 women in 2018 (Bray et al., 2018). It is still being the second most common cancer of Thai females (International Agency for Research on Cancer, 2018). The typical risk factor of cervical cancer is human papillomavirus (HPV) infection from early sexual intercourse; therefore patients’ age at diagnosis is younger than other cancers. Worldwide, the mean age of cervical cancer patients is 53 years with varying ages of 44-68 years from different countries (Arbyn et al., 2020). The majority of cervical cancer patients are diagnosed in locally advanced stage (stage IB3-IVA) with the range of percentage between 80-90% (Chirenje et al., 2000; Musa et al., 2016; Mlange et al., 2016; Sharma et al., 2017). Standard treatment for these stages is concurrent chemoradiation therapy (CCRT). Five-year overall survival (OS) rate was about 60% (Pearcey et al., 2002; Katanyoo et al., 2011). Because age at the beginning of disease in cervical cancer is in the working age, some adverse effects on their incomes and families negatively contributing to society are inevitable.

Cost of illness (COI) is an economic analysis that can reflect the economic burden from disease for healthcare provider, third-party payers, government and society (Hodgson, 1994; Luce et al., 1996; Rice, 2000). COI is necessary to define and prioritize health care policies for efficiency allocation health care resources and for selection of cost-effective interventions. However, there are limited evidences on cost of cervical cancer. According to the study from Japan evaluating the COI from governmental perspective during 1996 to 2011 (Hayata et al., 2015), COI had increased continuously, which derived from direct medical costs (DMCs) and mortality cost. Moreover, one study from Brazil reported that annual COI per patient was about US$ 2,000 based on a societal perspective (Santos et al., 2019). However, the generalizability of cost from one country to another is quite limited due to the difference of economic status and context.

The standard techniques of external beam radiation therapy (EBRT) which including two-dimensional technique (2D), three-dimensional conformal radiation therapy (3D-CRT), and intensity modulated radiation therapy (IMRT) are relatively high-cost. Nevertheless, outcomes of using 3D-CRT or IMRT were not superior to using 2D in terms of OS rate, but these modern techniques were able to reduce severe late side effects (Hasselle et al., 2011; Al Asiri et al., 2013).In Thailand, there were few studies which focused on economic analysis related to cervical cancer over the last decade (Suprasert and Manopunya, 2011; Termrungruanglert et al., 2012; Van Kriekinge et al., 2014). These included the burden of patients for follow-up program after treatment (Suprasert and Manopunya, 2011), cost of cervical cancer treatment using model to predict cost in provider perspective (Termrungruanglert et al., 2012), and the impact of HPV vaccination (Van Kriekinge et al., 2014). Nowadays, there is no study that reflects the burden of cervical cancer from a societal perspective, which derived from primary data. Therefore, the purpose of this study was to estimate the economic burden of locally advanced cervical cancer (LACC) Thai patients from a societal perspective. 

## Materials and Methods

*Study design*

This study employed an incidence-based approach from a societal perspective (Jo, 2014). The cost included direct medical costs (DMCs), direct non-medical costs (DnMCs) and indirect costs (IDCs). The time horizon in this study was 5 years from the treatment initiation. 

*Study sites and patients*

The study focused on the tertiary health facilities. Study sites were two tertiary hospitals i.e., Faculty of Medicine Vajira Hospital (teaching hospital) and the National Cancer Institute of Thailand (tertiary hospital), purposively selected based on accessibility of data. Study sample was new cervical cancer patients defined by the International Agency for Research on Cancer (IARC), the agency of the World Health Organization (WHO) in 2018 (International Agency for Research on Cancer 2018). All new cases during June to December 2019 were included. 

**Figure 1:**
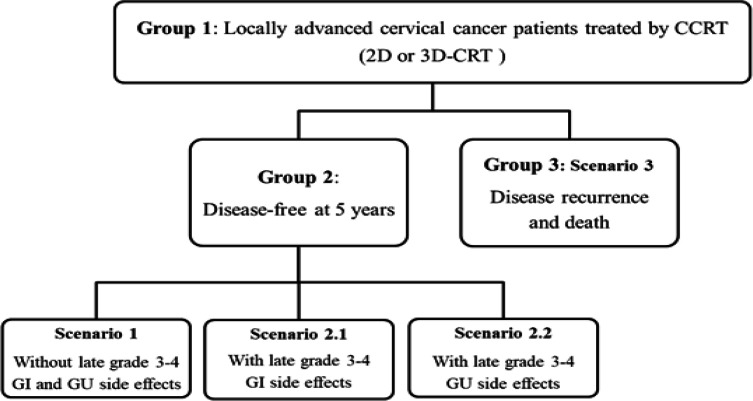
The Diagram of Three Common Scenarios and Three Health States of Patients. 2D, two-dimensional technique; 3D-CRT, three-dimensional conformal radiation therapy; GI, gastrointestinal; GU, genitourinary

LACC patients (stage IB3-IVA in FIGO version 2018) were divided into three groups as shown in Figure 1. The first group was patients who were receiving CCRT treatment at the time of interview. The second group was patients who had already completed treatment and were in follow-up program with no evidence of disease or disease-free at 5 years. The last group was patients who had completed treatment but had disease recurrence (local recurrence or distant metastasis). After the treatment, there were three probable scenarios: scenario1 -completely cured without severe late side effects; scenario 2- completely cured with grade 3-4 late side effects, which was classified to scenario 2.1- gastrointestinal (GI) side effects and scenario 2.2- genitourinary (GU) side effects; and scenario 3- recurrence of disease and death. The diagram of all scenarios was illustrated in Figure 1.

The study received ethical approval granted by the Institutional Review Board from these two hospitals and Mahidol University (Ref no 039/62, 044/2019 and 2019/030.1705). 

*Data collection and cost calculations*

The patients were interviewed to collect baseline characteristics (age, residence, education status, occupation, level of income per month and insurance scheme), DnMCs (travelling meal, hotel cost and time loss of caregivers) and IDCs (time loss of patients). All out-of-pocket expenses from treatments or disease since diagnosis of cervical cancer that incurred outside study sites were also examined. 

For cost calculations, costs of meal, transportation and accommodation were based on actual expenses from the interview. For caregiver time loss or informal care, opportunity cost method (Van den Berg et al., 2006) was employed. Cost per hour of caregiving time was referred to the Gross National Income (GNI) per capita of Thailand in 2019 from data of the World Bank; US $7,260 or 232,320 Thai Baht (THB) (The World Bank 2020) as the following formula (Riewpaiboon, 2014): 

Cost of informal care = Number of hour loss x productivity cost per hour

Productivity cost per hour = GNI per capita in 2019 / 52 (weeks per year) x 40 (working hour per week)

Indirect cost or cost of time loss or productivity loss of patients covered hospital-related absenteeism (morbidity cost) and premature mortality (mortality cost). The valuation of time loss was based on the human capital approach. Morbidity cost per person was calculated as follows:

Morbidity cost = Number of day loss x productivity cost per day

Productivity cost per day = GNI per capita in 2019 / 52 (weeks per year) x 5 (working day per week)

Regarding the mortality cost, the mean age of LACC patients which was used to be reference age in this study was 53 years (Arbyn et al., 2020). Median time to disease recurrence for symptomatic patients was varied from 14-36 months (Larson et al., 1988; Tinga et al., 1992; Morice et al., 2004; Sartori et al., 2007; Zola et al., 2007; Bodurka-Bevers et al., 2020). Therefore the mean age at death from cervical cancer by assumption was 56 years. Mortality cost was based on productivity loss from age at death until the year of official retirement of Thai people (60 years). The productivity loss was referred to the GNI per capita. Forecasted future GNI per capital was estimated based on 5% annual growth rate (Riewpaiboon, 2018). Present value was estimated using 3% discount rate (Permsuwan et al., 2014). The equation of cost of mortality per person was as follows ((Riewpaiboon, 2018) : 

Mortality cost = ∑i^n^Y_i_(1+r)^-i^

Y_i_ = average income per year at year of death to year of retirement (60 years)

n = total number of year from year of death to year of retirement (60 years) 

i = duration from first year to n

r = discount rate using 3% 

Regarding the cancer management, before starting treatment, all patients obtained complete staging including per vaginal examination, biopsy, chest x-ray and CT whole abdomen. For the treatment, CCRT comprised external beam radiation therapy (EBRT) in 28 fractions concurrence with weekly cisplatin 40 mg/m2 for six cycles. Four times of high dose-rate brachytherapy were applied nearly the end of EBRT. Follow-up program after finished treatment for all patients were per vaginal examination every three months at first two years, and then every six months to fifth years. Patients who had recurrence of disease had median overall survival 18 months by palliative chemotherapy as carboplatin plus paclitaxel for 6 cycles along with supportive and symptomatic treatment (Kitagawa et al., 2015). As there was a limited number of patients who completed treatment with grade 3-4 long-term GI and GU side effects (scenario 2.1 and 2.2), costs were estimated based on standard practice (Cashin and Ozaltin, 2014; Costing for UHC 2016).

Medical services received by the patients were extracted from hospital database of the study sites. Charges of all medical services received by the patients (DMCs) were based on 2019 prices and converted to costs using cost-to-charge ratio (cost/charge) of 1.63 (Chaikledkaew and Teerawattananon, 2013). 

All costs collected were in Thai baht in 2019 values. The presentation is in US dollar based on the exchange rate; USD 1 = 32 THB (Bank of Thailand 2020). 

E*stimation of the nationwide cost of locally advanced cervical cancer*

The estimation of the ratio between locally advanced stages (stage IB3-IVA in FIGO version 2018) and the other stages including early stages (stage IA-IB2 in FIGO version 2018) and metastatic stage (stage IVB), is 80:20 (Chirenje et al., 2000; Musa et al., 2016; Mlange et al., 2016; Sharma et al., 2017); thus 0.8 was multiplied by the estimated number of new cases. In 2018, Thai women aged 15-60 years which was the working age developed cervical cancer 5,567 patients, thus the estimated number of new cases in locally advanced stage was 4,450. 

The nationwide cost of LACC patients was obtained from COI per individual case of each scenario multiplied by total number of new cases and probabilities of each scenario. These probabilities from 2D were included overall survival (OS) rate, late grade 3-4 GI side effects, late grade 3-4 GU side effects and death rate at 5 years from the landmark study of Pearcy et al. (2002), which were 66%, 6%, 17% and 34%, respectively. For 3D-CRT, 2% of late grade 3-4 GI side effects and 7% of GU side effects were used from study of Hasselle et al., (2011). 

*Statistical and sensitivity analysis*

Data were managed by the MS Excel and then statistically analyzed using SPSS statistical software, version 22 (SPSS Inc., Chicago, IL). Descriptive statistics were used to describe basic characteristics. Age as a quantitative variable was presented as mean, standard deviation (SD), median and 95% confidence interval, while qualitative variables including residence, education status, occupation, level of income per month and payment scheme were presented as frequency and percentage. 

All costs were reported in terms of mean, SD, median and 95% confidence interval (95%CI). Cost data which were obtained using cross-sectional collection from patients in three health states were summation to be COI at 5 years for three scenarios. The total cost of economic burden from all LACC patients for 5 years of both 2D and 3D-CRT were derived from the summation of total costs from each scenario including no disease with no severe late side effects (scenario 1), no disease with severe late GI side effects (scenario 2.1), no disease with severe late GU side effects (scenario 2.2) and recurrence of disease and death (scenario3). Probabilistic sensitivity analysis (PSA) was applied to explore effect of variability of costs of each scenario on the total cost. Cost of each scenario was randomly selected in the range of 95% confidence interval. This Monte Carlo simulation was repeated for 1,000 times using in Microsoft Office Excel 2010 (Microsoft Corp., Redmond, WA). 

## Results

The LACC patients for three health states who were interviewed were 194. The mean and median age of all patients was 53 years (SD =11.4). However, mean and median age of patients who had disease recurrence was lesser than the other. Unemployment rate was higher among patients, who were in health state of disease recurrence. The basic characteristics are demonstrated in Table 1. 

For 5 year time horizon, the best scenario was disease-free without severe late side effects. The COI per one patient from societal perspective was US $8,391 and US $10,418 for using 2D and 3D-CRT, respectively. The majority cost was DMCs from cost of CCRT which accounted for 71% for 2D and 77% for 3D-CRT. DnMCs represented around 20%. On the other hand, productivity loss of patients or IDCs from cervical cancer was less than 10%, and was the smallest part in this scenario. The results are reported in Table 2.

The second scenario was disease-free with grade 3-4 of late GI or GU side effects. For this scenario, the results were separately reported by each side effect. DMCs of severe late GI and GU side effects were increased from no severe side effect about US $6,700 and US $8,500, respectively. With regard to severe late GI side effects, COI was US $18,018 for 2D and US $20,045 for 3D-CRT. While COI of severe late GU side effects for 2D and 3D-CRT was US $17,908 and US $19,936, respectively. Similarly, DMCs was the largest part, which accounted for about 70% for severe late GI side effects and 80% for severe late GU side effects. Details on COI of disease-free at 5 years with grade 3-4 of late GI side effects and GU side effects are demonstrated in Tables 3 and 4. 

The last scenario was disease recurrence and death which was the worst scenario of cervical cancer patients. Using the assumption of mean age at death from cancer was 56 years, COI of using CCRT as 2D and 3D-CRT was US $61,076 and US $63,103, respectively. The most influential cost was IDCs in terms of mortality cost which was taken part approximately 70% of COI. DMCs was accounted for 24% of the total cost, and 64% of DMCs was composed of the cost of palliative chemotherapy and supportive care. DnMCs contributed a small number of costs with lesser than 5% of the total COI. Cost of informal care was the most significant cost for this DnMCs. Premature death was the most important cost driver of COI accounted for 64 % of the total cost estimates. All results of this scenario were showed in Table 5. 

For the nationwide estimation, the cost of newly diagnosed 4,450 LACC patients in 2018 for 5 years was US $129 million and US $131 million for using 2D and 3D-CRT, respectively. The distribution of economic burden from each scenario including disease-free without severe late side effects, disease-free with grade 3-4 late GI side effects, disease-free with grade 3-4 late GU side effects and recurrence of disease and death was 19%, 2%, 7% and 72% for 2D, and 23%, 1%, 3% and 73% for 3D-CRT, respectively (Table 6). However, if the mean age at death was decreased to 50 years, the cost was increased of US $80 million to be US $209 million for 2D and US $212 million for 3D-CRT.

**Table 1:** Patient Characteristics Categorized by Groups of Health State

Characteristics	Receiving treatment (n=59) n (%)	Follow-up time with no disease (n=97) n (%)	Follow-up time with disease recurrence (n=38) n (%)
Age (mean ± SD/ median)	52.9 ± 11.9/ 53.0	55.1 ± 11.2/ 55.0	49.6 ± 10.7/ 47.5
Resident			
Bangkok	25 (42.4)	60 (61.9)	23 (60.5)
Outside Bangkok	34 (57.6)	37 (38.1)	15 (39.5)
Education status			
Uneducated	4 (6.8)	7 (7.2)	3 (7.9)
Primary school or below	32 (54.2)	56 (57.7)	20 (52.6)
Secondary school	18 (30.5)	19 (19.6)	8 (21.1)
Diploma	1 (1.7)	5 (5.2)	7 (18.4)
Bachelor or higher	4 (6.8)	10 (10.3)	0
Occupation			
Unemployed	23 (39.0)	36 (37.1)	28 (73.7)
Paid-employed	22 (37.3)	36 (37.1)	2 (5.3)
Self-employed	13 (22.0)	22 (22.7)	6 (15.8)
Government official	1 (1.7)	1 (1.0)	1 (2.6)
others	0	2 (2.1)	1 (2.6)
Average income per month ((US dollar in 2019)			
No income	23 (39.0)	76 (78.4)	24 (63.2)
< 156	6 (10.2)	21 (21.6)	2 (5.3)
156 - 313	11 (18.6)	0	3 (7.9)
314 -625	11 (18.6)	0	6 (15.8)
> 625	8 (11.6)	0	3 (7.9)
Payment scheme			
UCS	36 (61.0)	53 (54.6)	22 (57.9)
SSS	18 (31.5)	29 (29.9)	10 (26.3)
CSMBS	3 (5.1)	9 (9.3)	1 (2.6)
Self-pay	3 (5.1)	5 (5.2)	4 (10.5)
Disability	1 (1.7)	1 (1.0)	1 (2.6)

UCS, Universal Coverage Scheme; SSS, Social Security Scheme; CSMBS, Civil Servant Medical Benefit Scheme.

**Table 2:** Cost of Illness for Scenario 1: No Disease at 5 Years without Severe Late Side Effects (US dollar in 2019)

Classification of costs	Cost			95% confidence interval
	Mean	SD	Median	
Direct medical cost				
2D	5,985	120	5,118	5,978-5,993
3D-CRT	8,013	112	7,111	8,006-8,020
Direct non-medical cost	1,677	194	789	1,665-1,689
Travel	347	18	294	346-348
Food	221	24	166	219-222
Informal care	941	163	157	931-951
Indirect cost	729	28	639	727-730
COI using 2D	8,391	216	6,546	8,039-8,066
COI using 3D-CRT	10,418	214	8,539	10,067-10,093

**Table 3:** Cost of Illness for Scenario 2.1: No Disease at 5 Years with Severe Late Gastrointestinal Side Effects (US dollar in 2019)

Classification of costs	Cost			95% confidence interval
	Mean	SD	Median	
Direct medical cost				
2D	12,735	488	11,868	12,705-12,766
3D-CRT	14,763	488	10,695	14,733-14,793
Direct non-medical cost	3,763	188	2,072	3,751-3,775
Travel	766	28	506	764-767
Food	368	26	272	366-369
Informal care	2,466	164	157	2,456-2,476
Indirect cost	1,519	38	1,309	1,517-1,522
COI using 2D	18,018	541	15,249	17,984-18,052
COI using 3D-CRT	20,045	540	14,076	20,012-20,079

SD, standard deviation; 2D, two-dimensional technique; 3D-CRT, three-dimensional conformal radiation therapy

**Table 4:** Cost of Illness for Scenario 2.2: No Disease at 5 Years with Severe Late Genitourinary Side Effects (US dollar in 2019)

Classification of costs	Cost			95% confidence interval
	Mean	SD	Median	
Direct medical cost				
2D	14,544	515	13,676	14,512-14,576
3D-CRT	16,571	514	15,669	16,539-16,603
Direct non-medical cost	2,372	197	1,217	2,360-2,385
Travel	400	19	306	398-401
Food	238	24	172	237-240
Informal care	1,467	510	157	1,436-1,499
Indirect cost	992	29	902	990-994
COI using 2D	17,908	515	15,796	17,876-17,940
COI using 3D-CRT	19,936	514	17,788	19,904-19,967

SD, standard deviation; 2D, two-dimensional technique; 3D-CRT, three-dimensional conformal radiation therapy

**Table 5:** Cost of Illness for Scenario 3: Recurrence of Disease and Death (US dollar in 2019)

Classification of costs	Cost			95% confidence interval
Direct medical cost				
2D	16,546	514	14,733	16,515-16,578
3D-CRT	18,574	512	16,726	18,542-18,605
Direct non-medical cost	2,788	599	1,231	2,742-2,833
Travel	563	28	538	561-565
Food	351	27	344	350-353
Informal care	1,708	553	283	1,674-1,742
Indirect cost	41,742	323	40,775	41,726-41,765
Productivity loss	1,763	321	797	1,743-1,783
Mortality cost at 5 years	39,979	-	39,979	-
COI using 2D	61,076	861	56,739	61,023-61,129
COI using 3D-CRT	63,103	858	58,732	63,050-63,157

SD, standard deviation; 2D, two-dimensional technique; 3D-CRT, three-dimensional conformal radiation therapy

**Table 6:** Estimated of 5-Year Economic Burden from Newly Diagnosed Locally Advanced Cervical Cancer Patients in Thailand in 2018 (US dollar in 2019)

Scenarios	Cost			
	2D	%	3D-CRT	%
No disease with no severe late side effects	24,644,803	19	30,599,020	23
No disease with severe late GI side effects	3,175,132	2	1,177,462	1
No disease with severe late GU side effects	8,941,410	7	4,098,552	3
Recurrence of disease and death	92,408,033	72	95,475,356	73
Total cost	129,169,379	100	131,350,391	100

2D, two-dimensional technique; 3D-CRT, three-dimensional conformal radiation therapy; GI, gastrointestinal; GU, genitourinary

## Discussion

To the best of our knowledge, our study was the first study in Thailand that displayed the economic impact of LACC patients in terms of COI from a societal perspective. Due to different approach, different perspective and different time horizon from study of Termrungruanglert et al., (2012), all costs were unable to be compared. Nevertheless, the highest DMCs per one patient with stage IIB-IVA were reported comparing to other stages. That was confirmed the significant impact of these locally advanced stages on overall HPV-related diseases. Our study used the micro-costing or bottom-up method by obtaining all costs from real cost of each patient (Simoens, 2009). This method was recommended approaches more than macro approaches, because of more dependable and more precise (Mogyorosy and Smith, 2005). In 2019, one systematic review about the impact of costing methods for cervical cancer revealed the similar results between top-down and bottom-up method (Santos et al., 2019). The ratio between annual treatment cost of one cervical cancer patient and gross domestic product (GDP) per capita of top-down method and bottom-up method was 0.5:1 and 0.6:1, respectively. If the average of annual cost per patient in each scenario was done in our study and compared with GNI per capita of Thailand, the ratio were approximately 0.2:1 and 0.3:1 for scenario 1 by 2D and 3D-CRT, respectively, 0.5:1 for scenario 2 and 1.7:1 for scenario 3. The cost driver for scenario1 and scenario 2 was DMCs which represented approximately 70% and 80%, respectively. IDCs was the cost driver for scenario 3 and contributed 68% of the total cost.

The strength of our study was the comprehensive analysis for all common RT techniques as well as common scenarios in LACC patients for 5 years, particularly common severe side effects as GI and GU system from radiation therapy. If there was no these side effects, the lowest COI for 5 years was demonstrated. DMCs from using 3D-CRT was more expensive than 2D about US$2,000 per patient with increasing of six million dollar. However, when the common side effects in terms of GI and GU side effects were considered, COI of patients who had these events increased by two-fold. Using 3D-CRT conserved the total economic burden owning to lower probabilities of both side effects (Table 6). For the worst scenario, disease recurrence and death, all DMCs, DnMCs and IDCs were higher than the other scenario. For this scenario, IDCs accounted for the largest part of the COI. This consequence would raise catastrophic economic effect if age at death of cervical cancer was younger. 

Although cervical cancer can be effectively prevented through screening program and vaccination, the limitation of accessibility to such prevention is not surprising in underdeveloped and developing countries (Gakidou et al., 2008). When disease progressed to be in locally advanced stage, the economic burden was high due to cost of premature death. This finding was corresponded with Armenia, Azerbaijan, Belarus, Kazakhstan, Kyrgyzstan, the Russian Federation, Tajikistan, and Ukrain in Central Asia which found 1-6% increase every year in the number of premature death rate for patients aged younger than 50 years (Bray et al., 2013). That was conflicted with the study from Canada (Liu et al., 2016), which found that older age (>70 years) was a major factor affecting annual cost per case from the payer perspective. However, elderly patients might have co-morbidity, and that was also a cost driver in their study. Actually, these comorbid diseases could generate more cost instead of cancer.

However, this study has some limitations. Firstly, all costs related to severe late side effects were obtained from standard practice. It should be noted that there might be some missing data on DMCs and DnMCs, which were incurred by patients outside hospitals. In addition, a scenario in which patients have disease recurrence along with severe side effects was not included in our study. The next limitation was related to study sites, which were only located in Bangkok. Some DnMCs such as travel cost, accommodations or informal care might not reflect the cost of patients who received treatment in other provinces. The last limitation was related to the number of patients in the scenario of getting treatment as CCRT and disease recurrence. Due to the limited sample size, multiple regression analysis was not performed to examine the possible predictors for cost. Nevertheless, the major cost driver that our study was able to recognize was age at death. Death at early age would be resulted in the tremendous economic burden from their premature mortality. 

An empirical study for this economic analysis makes the recommendation regarding the actual expenses and time loss of patients and their families from cervical cancer. The real costs of these issues reflect the considerable burden outside the hospital which clinicians often ignore this problem. Holistic approach to overall health and financial burden in perspective of society is the important point for clinicians to attend. Moreover, a different cost from different treatment techniques versus different outcomes is the topic to pay attention in terms of cost effectiveness study before adopting them for routine practice.

To summarize, COI of locally advanced cervical cancer produced substantial economic burden for patients, their families and country from a societal perspective. Disease recurrence and early death from cancer was the most influential cause of this burden, which accounted for 70% of all total cost from all possible scenarios. The results of this economic analysis emphasized the importance of policy for prevention of invasive cancer including screening and vaccination in order to avoiding high cost from treatment and their sequelaes. Although 3D-CRT could reduce high cost of late complication treatment, but this technique produced higher overall cost than 2D. The results of this COI would be beneficial for further economic evaluation study.

## Author Contribution Statement

This article is a part of the thesis belong to the first author, which was approved for the Doctor of Philosophy of Social, Economic and Administrative Pharmacy (SEAP) Graduate Program, Faculty of Pharmacy, Mahidol University.

All authors contributed to the study conception and design. Data collection was conducted by KK. Data analysis was conducted by KK and AR. The first draft of the manuscript was written by KK. All authors commented on previous versions of the manuscript. All authors read and approved the final manuscript.
